# Decreased efficacy of an etonogestrel implant in a woman on antiepileptic medications: a case report

**DOI:** 10.1186/1752-1947-8-43

**Published:** 2014-02-11

**Authors:** Jill Lange, Stephanie Teal, Kristina Tocce

**Affiliations:** 1 Department of Obstetrics and Gynecology, University of Colorado Anschutz Medical Campus, 12631 E. 17th Avenue, Room 4006, Aurora, CO 80045, USA

## Abstract

**Introduction:**

Many antiepileptic drugs decrease the efficacy of combined hormonal contraceptives due to their inducing effect on cytochrome P450 liver metabolism. Less is known about the pharmacokinetics and outcomes of concomitant use of the etonogestrel implant and hepatic enzyme-inducing medications.

**Case presentation:**

A 22-year-old Hispanic woman with a long-standing seizure disorder treated with carbamazepine for 9 years became pregnant after 25 months of etonogestrel implant use.

**Conclusions:**

Hepatic enzyme-inducing drugs may reduce the efficacy of contraceptive implants. Contraceptive counseling for patients with medical co-morbidities requiring hepatic enzyme-inducing medications should include this information.

## Introduction

Hormonal contraceptives and antiepileptic drugs (AEDs) interact at the level of the liver’s cytochrome P450 (CYP450) enzyme pathway. Estrogens and progestins are metabolized by CYP450 3A4 [1-3]. Many AEDs, such as phenytoin, phenobarbital, oxcarbazepine, topiramate and carbamazepine are inducers of CYP450 3A4 isoenzymes in the liver [1]. Hepatic enzyme-inducing medications accelerate the metabolism of estrogen and progestins, thereby decreasing the intensity and duration of their action [1,2]. This phenomenon is best studied in patients using combined oral contraceptives (COCs) [2]. Carbamazepine co-administration with COCs results in decreased levels of contraceptive steroids, increased breakthrough bleeding and permits ovulation [3]. In a recent review of contraceptive methods and AEDs, 28 out of 34 studies evaluated COCs, and only four studied the levonorgestrel (LNG) contraceptive implant, which is no longer available in the USA [2].

Less is known about the widely used etonogestrel (ETG) implant (IMPLANON®, Merck, Darmstadt, Germany). In healthy women, the ETG implant is 99.95% effective [4]. Manufacturer’s trials report six pregnancies during 20,648 cycles, with each conception occurring shortly before or within 2 weeks after implant removal [5]. Women on medications that induce liver enzymes were excluded from the study. The implant package insert states that the ETG implant is not recommended for women requiring chronic use of inducing AEDs [5].

There are no published studies examining the pharmacokinetics of AEDs with the contemporary ETG implant. In the only study exclusively evaluating the pharmacokinetics of the LNG implant (Norplant), epileptic women treated with phenytoin alone or in combination with another anticonvulsant (*n*=6) were compared to healthy controls (*n*=10) and significantly lower levels of plasma LNG were observed in the group treated with AEDs at 3 to 12 months [6]. Two women with epilepsy (22%) became pregnant during the study period [2,6]. There are only three case series/reports documenting current ETG implant failure in women using AEDs [7-9]. Two case series evaluate multiple causes of failure: 1) Bensouda-Grimaldi *et al*. [7] document two out of 39 failures in France occurring in women using AEDs [7]; and 2) Harrison-Woolrych and Hill [8] report eight out of greater than 200 unintended pregnancies occurring with ETG implant and AED use [8]. Only one previous case report has exclusively documented a pregnancy in an ETG implant user on AEDs; this occurred 18 months after insertion [9]. This patient received an intrauterine device (IUD) following pregnancy termination due to the recognized potential interaction between AEDs and the implant [9].

We present a case in which a patient with a chronic AED history became pregnant after 25 months while using the ETG implant. She maintained the pregnancy, and had an uncomplicated term delivery. She then received another ETG implant postpartum at another institution.

## Case presentation

A 22-year-old gravid 2, para 0101, Hispanic woman with a long-standing history of a seizure disorder treated with carbamazepine 200mg four times a day for 9 years presented for ETG implant removal upon discovering she was pregnant. She suspected pregnancy secondary to late menses and morning sickness. She had the implant placed 25 months prior at the time of her LNG-intrauterine system (Mirena®, Bayer HealthCare, Wayne, NJ, USA) removal. She used the LNG-IUS for the preceding 30 months, which was placed at a 6-week post-partum visit, and discontinued it due to her partner’s discomfort during intercourse secondary to contact with the device strings. She had a pre-pregnancy body mass index of 29.2. An intrauterine pregnancy was confirmed with transvaginal ultrasound. The ETG implant was removed at that visit.

She was followed by maternal fetal medicine and neurology throughout her pregnancy. She experienced an increase in the number of seizures during her pregnancy, and her carbamazepine dose was increased to 400mg three times a day Her pregnancy was otherwise uncomplicated.

She delivered a healthy boy at 39 weeks, weighing 3.63kg (8lbs), by normal spontaneous vaginal delivery. At her 6-week post-partum visit at another institution, a second ETG implant was placed. There was no documentation of counseling regarding potential decreased efficacy of the implant due to her concomitant AED use. Despite the previous failure, she accepted another implant. She has not returned to our system or the institution that placed the second implant.

## Discussion

Patients who take medications that induce hepatic metabolism present challenges to providers prescribing hormonal contraception. Reliable contraception is essential for these women as many AEDs are teratogenic [1,3,10]. Studies have established significant decreases in COC effectiveness in women taking AEDs [2] and increasing COC doses to 50μg to overcome inducing effects of AEDs has been suggested [1,3,8]. Less is known about the pharmacokinetics of the ETG implant with hepatic enzyme-inducing AEDs, and providers must be aware of potential interactions. Certain AEDs are not inducers of hepatic metabolism and are safe and effective in combination with hormonal contraception. These include valproic acid, vigabatrin, lamotrigine, gabapentin, tiagabine, levetiracetam, zonisaminde, ethosuximide, and benzodiazepines [1,2,10]. When possible, these medications should be used for women with epilepsy who desire a full range of hormonal contraception options.

Providers should be aware of and utilize the *WHO Medical Eligibility Criteria for Contraceptive Use* (MEC). In women using hepatic enzyme-inducing AEDs, the MEC assigns a category 2 rating. (“Advantages generally outweigh theoretical or proven risks” [4]), and includes a clarifying comment: “Although the interaction of certain anticonvulsants with POPs and ETG implants is not harmful to women, it is likely to reduce the effectiveness of POPs and ETG implants” (POPs, progestogen-only pill) [4]. Long-term users of AEDs are specifically highlighted as an at-risk population for decreased implant efficacy: “Use of other contraceptives should be encouraged for women who are long-term users of any of these drugs” [4]. The failure of the ETG implant in our case report and one other both occurred in women with long-standing histories of treatment with inducing AEDs. Reliable contraceptive options assigned a category 1 rating (“A condition for which there is no restriction for the use of the contraceptive method” [4]) for hepatic enzyme-inducing AED users include LNG-IUD, copper IUD, and depot medroxyprogesterone acetate [4].

Studies mirroring the pharmacokinetic work with hepatic enzyme-inducing AEDs and COCs are needed to make evidence-based recommendations regarding the safety and efficacy of ETG implants in women taking these medications. Prior research investigating progestin implant metabolism in women using hepatic enzyme-inducing AEDs is limited by short study periods, a small number of women under investigation (N<20), incomplete information regarding AED type and dose, and its focus on an implant that is no longer available in the USA [2,6]. Pharmacokinetic and clinical trials are needed to quantify the effect of AEDs on ETG implant metabolism.

Until this evidence is available, it is prudent for patients and physicians to be aware of the potential interaction between the ETG implant and hepatic enzyme-inducing medications. In our case, the placement of a second implant despite its primary failure demonstrates a profound lack of awareness among providers. Although this issue is addressed in the Federal Drug Administration (FDA)-required training session for the ETG implant, providers are unlikely to retain all information distributed at these sessions. Further attention to this topic is needed in the literature.

## Conclusions

Medically complicated patients must receive focused contraceptive counseling regarding theoretical and known drug interactions. When determining individual method choice, ETG implants can be considered for women using hepatic enzyme-inducing AEDs if the risks and benefits are adequately addressed. With 99.95% efficacy, the implant is significantly more effective than other methods in women not using AEDs [4]. Until pharmacokinetic studies are performed that quantify the actual reduction in efficacy among these patients, patients can be counseled that efficacy may be decreased, but is still likely to be higher than barrier methods, progestin-only pills, and COCs. High-efficacy contraceptive alternatives, or AEDs that do not interact with hepatic metabolism, should be considered.

## Consent

Written informed consent was obtained from the patient for publication of this case report. A copy of the written consent is available for review by the Editor-in-Chief of this journal.

## Abbreviations

AEDs: Antiepileptic drugs; COCs: Combined oral contraceptives; CYP450: Cytochrome P450; ETG: Etonogestrel; IUD: Intrauterine device; IUS: Intrauterine system; LNG: Levonorgestrel; MEC: *WHO Eligibility Criteria for Contraceptive Use*; POPs: Progestogen-only pills.

## Competing interests

The authors declare that they have no competing interests.

## Authors’ contributions

JL performed the literature review, draft writing and editing, as well as manuscript submission and revision. ST contributed to idea conception as well as editing manuscript drafts. KT contributed to idea conception and execution, draft writing, and was a major contributor to manuscript editing. All authors read and approved the final manuscript.
